# Fingerprinting of hatchery haplotypes and acquisition of genetic information by whole-mitogenome sequencing of masu salmon, *Oncorhynchus masou masou*, in the Kase River system, Japan

**DOI:** 10.1371/journal.pone.0240823

**Published:** 2020-11-04

**Authors:** Yoko Kato-Unoki, Keitaro Umemura, Kosuke Tashiro

**Affiliations:** 1 Center for Advanced Instrumental and Educational Supports, Faculty of Agriculture, Kyushu University, Fukuoka, Japan; 2 Fishery Research Laboratory, Kyushu University, Fukuoka, Japan; 3 Laboratory of Molecular Gene Technology, Faculty of Agriculture, Kyushu University, Fukuoka, Japan; National Cheng Kung University, TAIWAN

## Abstract

Stocking hatchery fish can lead to disturbance and extinction of the local indigenous population. Masu salmon *Oncorhynchus masou masou*, which is endemic across Japan, is a commonly stocked fish for recreational fishing in Japan. To conserve the indigenous resource, their genetic information is required, however, especially on Kyushu Island, the paucity of genetic information for this species has hindered proper resource management. Here, to identify hatchery mitogenome haplotypes of this species, stocked in the Kase River system, Kyushu Island, Japan, and to provide mitogenomic information for the resource management of this species, we analyzed the whole-mitogenome of masu salmon in this river system and several hatcheries potentially used for stocking. Whole-mitogenome sequencing clearly identified hatchery haplotypes, like fingerprints: among the 21 whole-mitogenome haplotypes obtained, six were determined to be hatchery haplotypes. These hatchery haplotypes were distributed in 13 out of 17 sites, suggesting that informal stocking of *O*. *m*. *masou* has been performed widely across this river system. The population of no hatchery haplotypes mainly belonged to clade I, a clade not found in Hokkaido Island in previous studies. Sites without hatchery haplotypes, and the non-hatchery haplotypes in clade I might be candidates for conservation as putative indigenous resources. The whole-mitogenome haplotype analysis also clarified that the same reared strain was used in multiple hatcheries. Analysis of molecular variance suggested that stocked hatchery haplotypes reduce the genetic variation among populations in this river system. It will be necessary to pay attention to genetic fluctuations so that the resources of this river system will not deteriorate further. The single nucleotide polymorphism data obtained here could be used for resource management in this and other rivers: e.g., for monitoring of informal stocking and stocked hatchery fishes, and/or putative indigenous resources.

## Introduction

Stocking of hatchery fish has been carried out throughout the world to mitigate declines in natural production and enhance fishery production and recreational fishing [1]. However, it can lead to disturbance and extinction of the local indigenous population, and loss of genetic diversity [2, 3]. Such effects are observed among salmonid fishes (e.g., [4–6]).

Masu salmon, *Oncorhynchus masou masou* is endemic across Japan, with diverse life histories [7]: there is a river-resident form and an anadromous form [7], with spawning adults homing to their natal river at high rates, similar to other Pacific salmons [8]. *Oncorhynchus masou masou* is one of the commonly stocked fish for recreational fishing in Japan. Especially on Kyushu Island, public resource management is not sufficient in many rivers, and inappropriate and informal stocking is performed widely by individuals and/or fishing clubs. Recent research has suggested that *O*. *m*. *masou* can be divided into different populations in terms of behavior, morphology, and genetics in each river [9–12]. Stocking hatchery *O*. *m*. *masou* might reduce the indigenous characteristics and local genetic variation, as observed in other salmonid species (e.g., [4–6]). To conserve and properly manage the indigenous *O*. *m*. *masou* resource in a river, their genetic information is required. Genetic study of *O*. *m*. *masou* in Japan has focused mainly on Hokkaido Island, and some areas of Honshu Island and its surrounding waters (e.g., [9, 12–14]); studies of this species in Kyushu Island are scarce [15, 16].

**Fig 1:**
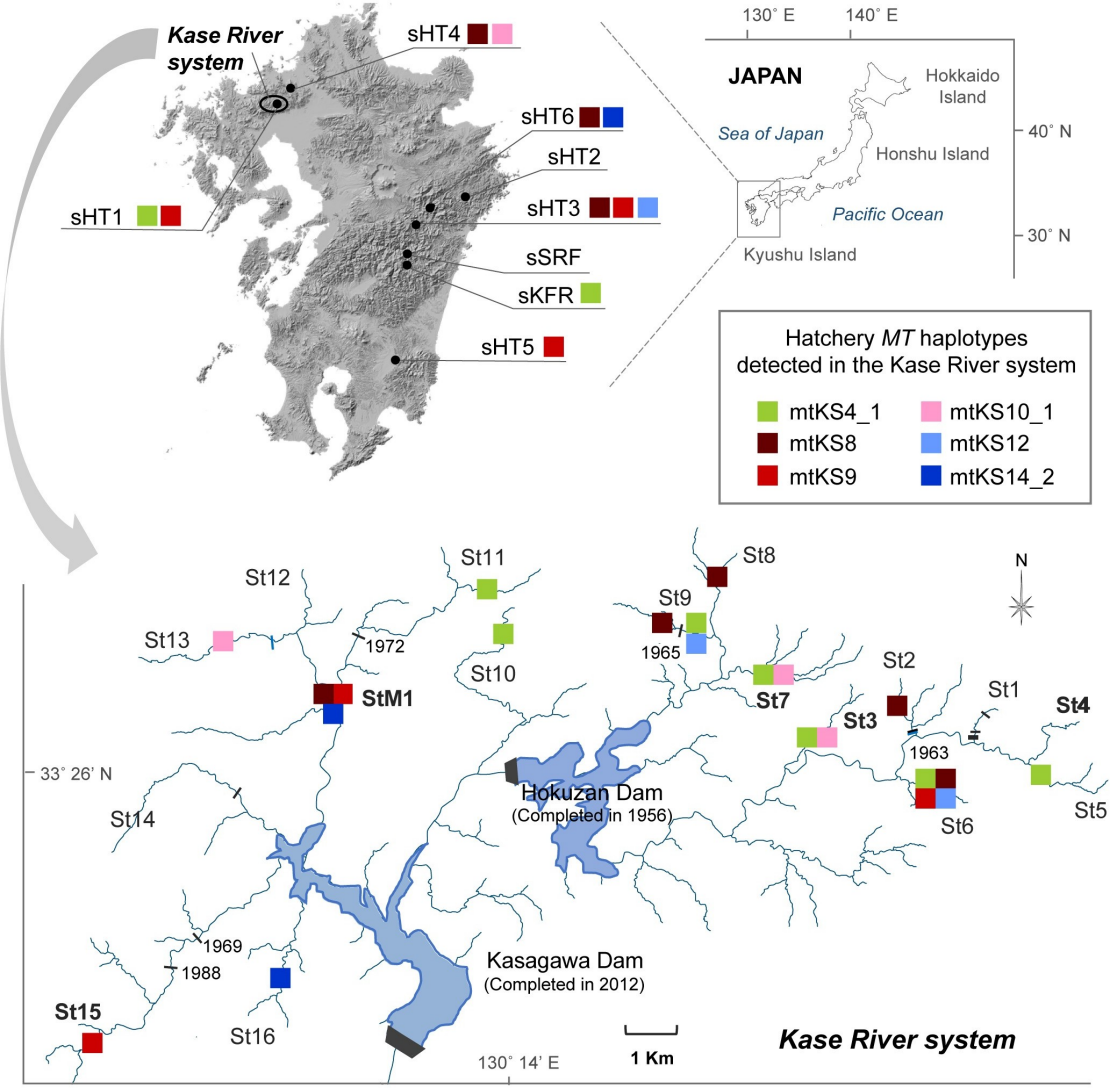
Sampling locations and hatchery whole-mitogenome (*MT*) haplotype distributions detected in the Kase River system. The upper map shows Kyushu Island, and the lower map shows the Kase River system. In the lower map, the main man-made dams (black bars with build year) and waterfalls (blue bars) that may hinder the fish run are marked. Sites with prior information about stocking history are shown in bold letters. For details of the samples and the results of whole-mitogenome sequencing, see Tables 1 and 4. The upper left map was modified from data downloaded from the Geospatial Information Authority of Japan (GSI) (original copyright 2020), and the upper right map and lower map were modified from data downloaded from the Geographic Information System (GIS) web page of the National Land Information Division, Japan (original copyright 2007) by the authors, respectively.

The Kase River system is located in northwestern Kyushu Island (Fig 1). Inappropriate *O*. *m*. *masou* stocking, with no consideration of the indigenous population, has been conducted for a long time by fishing clubs and personal game fishers in this river. However, there have been no investigations of this species in this river system, and the distributions of foreign genes (hereafter, “hatchery haplotypes”) introduced by stocking and information of indigenous haplotypes are unknown. The lack of these genetic information hinders resource management of this species in this river system.

Whole-mitogenome analysis will provide useful genetic information of *O*. *m*. *masou*. The mitogenome is commonly used as a genetic marker for characterizing population structure and identifying maternal lineages [17–19], and is also used as the marker for environmental DNA (eDNA) analysis, which is recently attracting attention in resource management [20–22]. Recent reports suggest that whole-mitogenome analysis can increase the resolution of matrilineal genetic patterns, especially in low diversity species, and allow detailed and more accurate phylogenetic analysis (e.g., [23–26]). Accordingly, although high-resolution single nucleotide polymorphism (SNP) information might be required to distinguish subtle differences between similar lineages in a species, whole-mitogenome analysis would provide sufficient information for this species, and a stocked haplotype in the field could be identified by using the complete mitogenome sequence used for stocking as a reference.

Here, to identify hatchery mitogenome haplotypes, and to provide genetic information for the resource management of *O*. *m*. *masou* in the Kase River system, we analyzed the whole-mitogenome of this species in this river system and in several hatcheries that might be used for stocking. Through this analysis, we clarified the distribution of hatchery haplotypes and provided information regarding “other haplotypes” (probably including indigenous haplotypes) in this river system. Obtained information and the SNP data will contribute to resource management in this and other river systems.

## Materials and methods

### Ethics statement

This study was conducted with the permission of the Saga prefecture (permission number 3018). The fish were collected under appropriate fishing licenses that allowed the capture and sacrifice of the fish. The ethical approvals of the Kyushu University Animal Experiment Committee and the Faculty of Agriculture Ethics Committee in the Kyushu University were not required because approval of animal experiments is only necessary for fish reared at Kyushu University (according to the Kyushu University Animal Experimentation Regulations) and ethics covers only human research (this study is fish research) (according to the Faculty of Agriculture Ethics Committee Regulations in the Kyushu University). However, this study was carried out according to the guidelines of the Ichthyological Society of Japan (http://www.fish-isj.jp/english/guidelines.html).

### Study area and samples

Information about the *O*. *m*. *masou* samples (geographic coordinates, sample numbers, sampling year, and stocking record) used in this study is listed in Table 1, and the locations of sampling sites are shown in Fig 1. The samples from the Kase River system were collected at 16 sites (St1–St16)—a single site on each of 16 tributaries—and at StM1, which is on tributary 11 at the confluence of tributaries 11 and 12, and a well-known as a stocking site (Fig 1 and Table 1). Samples from Shiiba Research Forest, Kyushu University (sSRF), an unstocked site in the Hitotsuse River system, were used as the control group. Information on the stocking record and hatcheries used for the Kase River system were collected, and one research center (Miyazaki Prefectural Fisheries Research Institute, sKFR) and six hatcheries (sHT1–sHT6) were selected as putative sources of *O*. *m*. *masou* in this river system (Fig 1 and Table 1) [16, 27]. Information about the river of origin and mating history of hatchery fishes is shown in the columns “River system / tributary” and “Additional information”, respectively, in Table 1.

**Table 1:** Information about samples used in this study.

	Site ID	River system / tributary^a^	Latitude / Longitude	Sampling year	Number of samples	Stocking record	Additional information
**Kase River system**	St1	Kase / tributary 1	33.44 / 130.32	2016	16	unknown	
	St2	Kase / tributary 2	33.44 / 130.30	2016	21	unknown	
	St3	Kase / tributary 3	33.43 / 130.29	2016	5	stocked	
	St4	Kase / tributary 4	33.43 / 130.33	2015, 2016	23	stocked	
	St5	Kase / tributary 5	33.43 / 130.33	2016	21	unknown	
	St6	Kase / tributary 6	33.43 / 130.31	2016	12	unknown	
	St7	Kase / tributary 7	33.44 / 130.28	2016	8	stocked	
	St8	Kase / tributary 8	33.46 / 130.27	2016	6	unknown	
	St9	Kase / tributary 9	33.45 / 130.26	2016	33	unknown	
	St10	Kase / tributary 10	33.45 / 130.23	2016	26	unknown	
	St11	Kase / tributary 11	33.46 / 130.23	2016	22	unknown	
	St12	Kase / tributary 12	33.47 / 130.20	2016	20	unknown	
	St13	Kase / tributary 13	33.45 / 130.18	2016	16	unknown	
	St14	Kase / tributary 14	33.43 / 130.17	2016	11	unknown	
	St15	Kase / tributary 15	33.38 / 130.15	2016	11	stocked	
	St16	Kase / tributary 16	33.40 / 130.19	2016	11	unknown	
	StM1	Kase / tributary 11	33.44 / 130.20	2016	24	stocked	
**Control**	sSRF	Hitotsuse	32.28 / 131.13	2016	20	unstocked	prohibited fishing area
**Hatchery etc.**	sKFR	Hitotsuse / Ishido	33.37 / 131.14	2016	7	-	from native fish [16]
	sHT1	Hitotsuse / Ishido	33.43 / 130.32	2016	11	-	from sKFR
	sHT2	Kita	32.80 / 131.63	2016	15	-	from native fish [27]
	sHT3	Gokase	32.58 / 131.15	2016	15	-	mated with native fish and others [16]
	sHT4	-	33.49 / 130.50	2016	10	-	mated with many strain
	sHT5	Ooyodo / Okimizu	31.74 / 131.23	2016	10	-	from native fish [16]
	sHT6	Gokase / Kawabashiri	32.81 / 131.20	2016	9	-	mated with native fish and others

^a^For each hatchery, the original river of the reared fish is given.

### Sampling and DNA extraction

Wild fish were captured by electrofishing or normal fishing from 2015 to 2016. A small piece of fin was taken from each specimen, and the captured fish were released at the same point in the river. Tissues were immediately fixed in 99.5% ethanol or RNA*later* solution (Thermo Fisher Scientific) and stored at −20°C until use. Total genomic DNA was extracted from preserved tissue using the QIAGEN Blood & Tissue DNeasy Kit (QIAGEN).

### DNA sequencing

Next-generation sequencing (NGS) analysis is still expensive. To limit the sample number required for NGS, we performed whole-mitogenome analysis for some of the samples of each haplotype from each site (88 individuals) after comprehensively obtaining the haplotypes of all samples (383 individuals) by partial mitogenome sequencing. The region of partial sequencing was the NADH dehydrogenase subunit 5 gene (*ND5*), which has been used in previous studies in this species [9, 13].

The *ND5* region (1597 bp) was amplified with the primer pair ND5-F1 and ND5-R (Table 2). The PCR mixture was as follows: 10–50 ng genomic DNA, 1× Phusion HF buffer (New England BioLabs), 0.2 mM of each dNTP, 0.5 μM of each primer, and 0.1 μL Phusion DNA polymerase (New England BioLabs) in a total volume of 10 μL. The PCR program was as follows: one cycle at 98°C for 30 s, followed by 40 cycles of 98°C for 10 s, 64°C for 20 s, and 72°C for 60 s, and a final extension at 72°C for 2 min. Each PCR product was sequenced with the primer ND5-F1 or ND5-F2 (Table 2). The *ND5* sequences were assembled and multiple sequences were aligned using ATGC Ver. 4.3.5 software (GENETYX Co.). The identified 1449 bp sequence variants (*ND5* haplotypes) (*ND5* position, 358–1806; mitogenome position, 13299–14747 for reference sequence NC_008747) were deposited in DDBJ/EMBL/GenBank as shown in S1 Table. The ND5 sequencing procedures was deposited at protocols.io. (dx.doi.org/10.17504/protocols.io.bmf7k3rn).

**Table 2:** List of primers used in this study.

Name	Sequence (5ʹ–3ʹ)	Primer position^a^	
		mitogenome	*ND5*
ND5-F1^b^	TACCCCAATTGCCCTGTACG	13223–13242	282–301
ND5-F2	TCAGGCTCAATTATCCACAG	13986–14005	1045–1064
ND5-R^b^	ACTAACACGTGGGTTAGGTCGAG	14797–14819	(3ʹ NCR)^c^
MT-F	AATTATCCACAGTTTAAATGACGAACAAG	13994–14022	1053–1081
MT-R	AAAAGTATAGCTTTAAAGAATGCGTGAGT	13950–13978	1009–1037

^a^The primer is positioned in the reference sequence (NC_008747).

^b^Reference taken from Kitanishi et al. [9]; however, ND5-R varied slightly from this reference.

^c^3ʹ terminal non-coding region (3ʹ NCR) of *ND5*.

The whole-mitogenome fragment (16.6 kbp) was amplified with primer pair MT-F and MT-R (Table 2). The PCR mixture was as follows: 20–100 ng genomic DNA, 1× PrimeSTAR GXL Buffer (TaKaRa Bio Inc.), 0.2 mM each dNTP, 0.2 μM each primer, and 0.3 μL of PrimeSTAR GXL DNA polymerase (TaKaRa Bio Inc.) in a total volume of 15 μL. The PCR program was as follows: one cycle at 98°C for 10 s, followed by 40 cycles of 98°C for 10 s, 60°C for 15 s, 68°C for 14 min, and a final extension at 68°C for 5 min. Each PCR product was subjected to agarose gel electrophoresis, and the mitogenome was extracted from the excised band by using the Wizard SV Gel and PCR Clean-up System (Promega).

Mitogenome libraries for NGS were prepared using a QIAseq FX DNA Library Kit (QIAGEN). For each library, the quality and fragment size were checked using an Agilent BioAnalyzer and the concentration was quantified by qPCR (Mx3000p, Agilent) using a KAPA Library Quantification Kit (KAPA Biosystems). Each library was pooled in equal amounts to obtain the final library. The pooled and denatured library (8 pM) containing 5% volume of PhiX (control library; Illumina) was sequenced using the Illumina MiSeq system with MiSeq Reagent Kit V3 (300 bp paired-end reads) (Illumina).

The obtained NGS data of paired end reads were trimmed using Trimmomatic ver. 0.36 as follows: a cleanup adapter was applied and reads with low quality (Q score, <28) and short-length (<50) were filtered out; then, the reads were mapped to the reference mitogenome sequence (*O*. *m*. *masou* accession No: NC_008747) using Burrow–Wheeler Aligner ver. 0.7.12, and the obtained SAM (Sequence Alignment Map) files were converted to BAM (Binary Alignment Map) files using SAMtools ver. 1.4.1. The resulting reads and the SNP sites for the reference sequence were visualized with Integrative Genomics Viewer version 2.3.83. and TASSEL ver. 5.0. The primer sites and those outside the region of interest (mitogenome positions 13950–14022) were replaced with the predetermined *ND5* sequence. The identified full-length mitogenome sequence variants (*MT* haplotypes) were deposited in DDBJ/EMBL/GenBank as shown in S1 Table. Representative FASTQ data of each *MT* haplotypes were deposited in DRA (DDBJ Sequence Read Archive) under the accession number DRA007284. The whole-mitogenome sequencing procedures were deposited at protocols.io. (dx.doi.org/10.17504/protocols.io.bkwykxfw; dx.doi.org/10.17504/protocols.io.bmgck3sw).

### Data analyses

Haplotype diversity and nucleotide diversity at each site in the Kase River were quantified by using Arilequin v3.5 [28]. Genetic variation in the Kase River system among the populations (corresponding to Site IDs, Table 1) or within the population (Analysis 1) and “other haplotypes” (excluding hatchery haplotype samples; Analysis 2) were calculated by analysis of molecular variance (AMOVA) implemented in Arilequin v3.5 based on the *ND5* haplotype data (Table 3). The population of StM1, which is the confluence of tributaries 11 and 12, was excluded from both analyses. Samples that did not match with a hatchery haplotype in whole-mitogenome analysis (i.e., samples of mtKS4_2 at St8, mtKS6_1 at St9, and mtKS14_1 at St14) were added to Analysis 2.

**Table 3:** Distribution of *ND5* haplotypes at each sampling site.

		KS1	KS2	KS3	KS4*	KS5	KS6*	KS7	KS8*	KS9*	KS10*	KS11	KS12*	KS13	KS14*	KS15	HT3*	HT4A*	HT4B*	Total (n)	Haplotype diversity	Nucleotide diversity
**Kase River system**	**St1**	0	0	16	0	0	0	0	0	0	0	0	0	0	0	0				16	0.0000	0.0000
	**St2**	0	0	2	0	0	0	0	19	0	0	0	0	0	0	0				21	0.1810	0.0012
	**St3**	0	0	2	1	0	0	0	0	0	2	0	0	0	0	0				5	0.8000	0.0057
	**St4**	5	12	0	0	0	0	0	0	0	0	0	0	0	0	6				23	0.6403	0.0026
	**St5**	2	14	0	5	0	0	0	0	0	0	0	0	0	0	0				21	0.5143	0.0007
	**St6**	0	0	0	7	0	0	0	1	3	0	0	1	0	0	0				12	0.6364	0.0045
	**St7**	0	0	0	7	0	0	0	0	0	1	0	0	0	0	0				8	0.2500	0.0022
	**St8**	0	0	1	1	0	0	0	4	0	0	0	0	0	0	0				6	0.6000	0.0038
	**St9**	0	0	3	2	0	2	0	20	0	0	4	2	0	0	0				33	0.6174	0.0031
	**St10**	0	0	7	19	0	0	0	0	0	0	0	0	0	0	0				26	0.4092	0.0006
	**St11**	0	0	8	14	0	0	0	0	0	0	0	0	0	0	0				22	0.4848	0.0007
	**St12**	0	0	0	0	20	0	0	0	0	0	0	0	0	0	0				20	0.0000	0.0000
	**St13**	0	0	3	0	0	0	0	0	0	1	0	0	5	0	7				16	0.7167	0.0029
	**St14**	0	0	0	0	0	0	2	0	0	0	0	0	0	9	0				11	0.3273	0.0014
	**St15**	0	0	0	0	0	0	0	0	11	0	0	0	0	0	0				11	0.0000	0.0000
	**St16**	0	0	0	0	0	0	0	0	0	0	0	0	0	1	10				11	0.1818	0.0001
	**StM1**	0	0	2	0	1	0	8	2	2	0	0	0	1	8	0				24	0.7862	0.0037
	**Total (n)**	7	26	44	56	21	2	10	46	16	4	4	3	6	18	23				286		
**Control**	**sSRF**	0	0	0	20	0	0	0	0	0	0	0	0	0	0	0	0	0	0	20		
**Hatchery etc.**	**sKFR**	0	0	0	7	0	0	0	0	0	0	0	0	0	0	0	0	0	0	7		
	**sHT1**	0	0	0	6	0	0	0	0	5	0	0	0	0	0	0	0	0	0	11		
	**sHT2**	0	0	0	0	0	0	0	0	0	15	0	0	0	0	0	0	0	0	15		
	**sHT3**	0	0	0	0	0	3	0	1	4	2	0	3	0	1	0	1	0	0	15		
	**sHT4**	0	0	0	0	0	1	0	2	0	1	0	0	0	0	0	1	1	4	10		
	**sHT5**	0	0	0	0	0	0	0	1	5	2	0	0	0	0	0	2	0	0	10		
	**sHT6**	0	0	0	0	0	0	0	2	0	0	0	0	0	4	0	1	2	0	9		
	**Total (n)**	0	0	0	13	0	4	0	6	14	20	0	3	0	5	0	5	3	4	77		

Asterisked haplotypes indicate the hatchery haplotype in *ND5* haplotype.

The relationships among haplotypes were estimated using the TCS program implemented in PopART [29]. To determine the relationships between haplotypes in past studies and the current study, we also analyzed the haplotype network including data from Kitanishi et al. [9] and Yu et al. [13].

## Results

### DNA sequences and determination of haplotype

The sequencing analysis of the 1449 bp fragment containing the *ND5* gene detected a total of 18 *ND5* haplotypes with 28 SNP sites (SNP position 13430–14687 in S2 Table) from 383 individuals (Table 3). Of these, 10 *ND5* haplotypes (KS4, KS6, KS8–KS10, KS12, KS14, HT3, HT4A, and HT4B) were detected in hatcheries; seven of these 10 *ND5* hatchery haplotypes (KS4, KS6, KS8–KS10, KS12, and KS14) were detected in the Kase River system. KS4 was also detected in the unstocked area, sSRF, in the Hitotsuse River system.

Eighty-eight of the 383 individuals that underwent *ND5* sequencing were selected for whole-mitogenome sequencing by NGS. The whole-mitogenome sequencing of all 88 individuals was successful with approximately >1500-fold depth of coverage. The numbers of samples with each determined *MT* haplotype are shown for each sampling site in Table 4. The majority of *ND5* haplotypes were associated with more than one *MT* haplotype, with 32 haplotypes including 256 SNP sites being detected (Table 4 and S2 Table). Sixteen haplotypes were detected in hatcheries (see asterisks, Table 4), and six of these, mtKS4_1, mtKS8, mtKS9, mtKS10_1, mtKS12, and mtKS14_2, were detected in the Kase River system. Hatchery haplotypes were distributed in 13 sites including sites with unknown stocking record (Fig 1). Several hatchery haplotypes were detected upstream of artificial barriers (e.g., dams) and/or natural barriers (e.g., waterfalls): mtKS4_1 at St11; mtKS8 at St2 and St9; mtKS9 at St15; and mtKS10_1 at St13 (Fig 1). The whole-mitogenome sequences of hatchery *ND5* haplotype KS6 in the Kase River system (mtKS6_1) and KS4 at sSRF (mtKS4_HIT) did not match any hatchery *MT* haplotypes in the samples analyzed here (Table 4). Two *MT* haplotypes, mtKS4_1 and mtKS9, were detected in hatchery sHT1, which reared fish from sKFR; one of these *MT* haplotypes, mtKS4_1, was the same as that detected in sKFR (Tables 1 and 4). The *MT* haplotype determined for the *ND5* haplotype KS4 samples in the Kase River system was consistently mtKS4_1 except for a sample at St8, which was mtKS4_2.

**Table 4:** Results of whole-mitogenome sequencing.

		KS	KS		KS			KS			KS	KS		KS	KS	KS	KS			KS	KS	KS		KS				KS	HT3*			HT4A*	HT4B*	Total (n)
		1	2		3			4*			5	6*		7	8*	9*	10*			11	12*	13		14*				15						
mtKS1	mtKS2_1	mtKS2_2	mtKS3_1	mtKS3_2	mtKS3_3	mtKS4_1*	mtKS4_2	mtKS4_HIT	mtKS5	mtKS6_1	mtKS6_HT3*	mtKS7	mtKS8*	mtKS9*	mtKS10_1*	mtKS10_HT2*	mtKS10_HT3*	mtKS11	mtKS12*	mtKS13_1	mtKS13_2	mtKS14_1	mtKS14_2*	mtKS14_HT3*	mtKS14_HT6*	mtKS15	mtHT3_1*	mtHT3_HT4*	mtHT3_HT6*	mtHT4A*	mtHT4B*			
**Kase River system**	**St1**				4																													4
	**St2**					1									1																			2
	**St3**				1			1									1																	3
	**St4**	1	1																									1						3
	**St5**	1		3				1																										5
	**St6**							1							1	1					1													4
	**St7**							1									1																	2
	**St8**				1				1						1																			3
	**St9**				1			1				1			2					1	1													7
	**St10**				1			2																										3
	**St11**						2	1																										3
	**St12**										4																							4
	**St13**						1										1					1						1						4
	**St14**													1										1										2
	**St15**															1																		1
	**St16**																								1			1						2
	**StM1**										1			1	1	1							1		1									6
**Control**	**sSRF**									1																								1
**Hatchery** **etc.**	**sKFR**							1																										1
	**sHT1**							1								1																		2
	**sHT2**																	1																1
	**sHT3**												1		1	1			1		1					1			1					7
	**sHT4**												1		1		1													1		1	1	6
	**sHT5**															3			2															5
	**sHT6**														1										1		3				1	1		7
***MT* haplotype (n)**		2	1	3	8	1	3	10	1	1	5	1	2	2	9	8	4	1	3	1	3	1	1	1	3	1	3	3	1	1	1	2	1	88
***ND5* haplotype (n)**		2	4		12			12			5	3		2	9	8	8			1	3	2		8				3	3			2	1	88

For each site, the number of samples with the indicated genotype is presented. Asterisked haplotypes indicate the hatchery haplotype.

Some hatchery haplotypes were detected at multiple hatcheries (Table 4): mtKS6_HT3 was detected at sHT3 and sHT4; mtKS8 was detected at sHT3, sHT4, and sHT6; mtKS9 was detected at sHT1, sHT3, and sHT5; mtKS10_HT3 was detected at sHT3 and sHT5; and mtHT4A was detected at sHT4 and sHT6.

### Data analyses

Based on the *ND5* sequences (Table 3), haplotype diversity ranged from 0.000 to 0.8000 and nucleotide diversity from 0.0000 to 0.0057 (Table 3). The molecular variance within the population of “other haplotype” samples (Analysis 2) was 33.02%, which was less than that of all samples (44.15%) (Analysis 1). The molecular variance among populations when hatchery haplotype samples were excluded (Analysis 2) was 66.98%, which is higher than that when all samples were included (55.85%) (Analysis 1) (Table 5).

**Table 5:** Analysis of molecular variance (AMOVA) based on the *ND5* sequences in the Kase River system.

Analysis	Source of variation	d.f.	Sum of squares	Variance components	Percentage of variation
1) All samples^a^	Among populations	15	64.615	0.25439	55.85**
	Within populations	246	49.476	0.20112	44.15**
	Total	261	114.091	0.45551	
2) Samples with “other haplotypes”	Among populations	12	35.803	0.29099	66.98**
	Within populations	119	17.068	0.14343	33.02**
	Total	131	52.871	0.43442	

^a^Includes samples with hatchery haplotypes and those with other haplotypes.

**p < 0.01.

**Fig 2:**
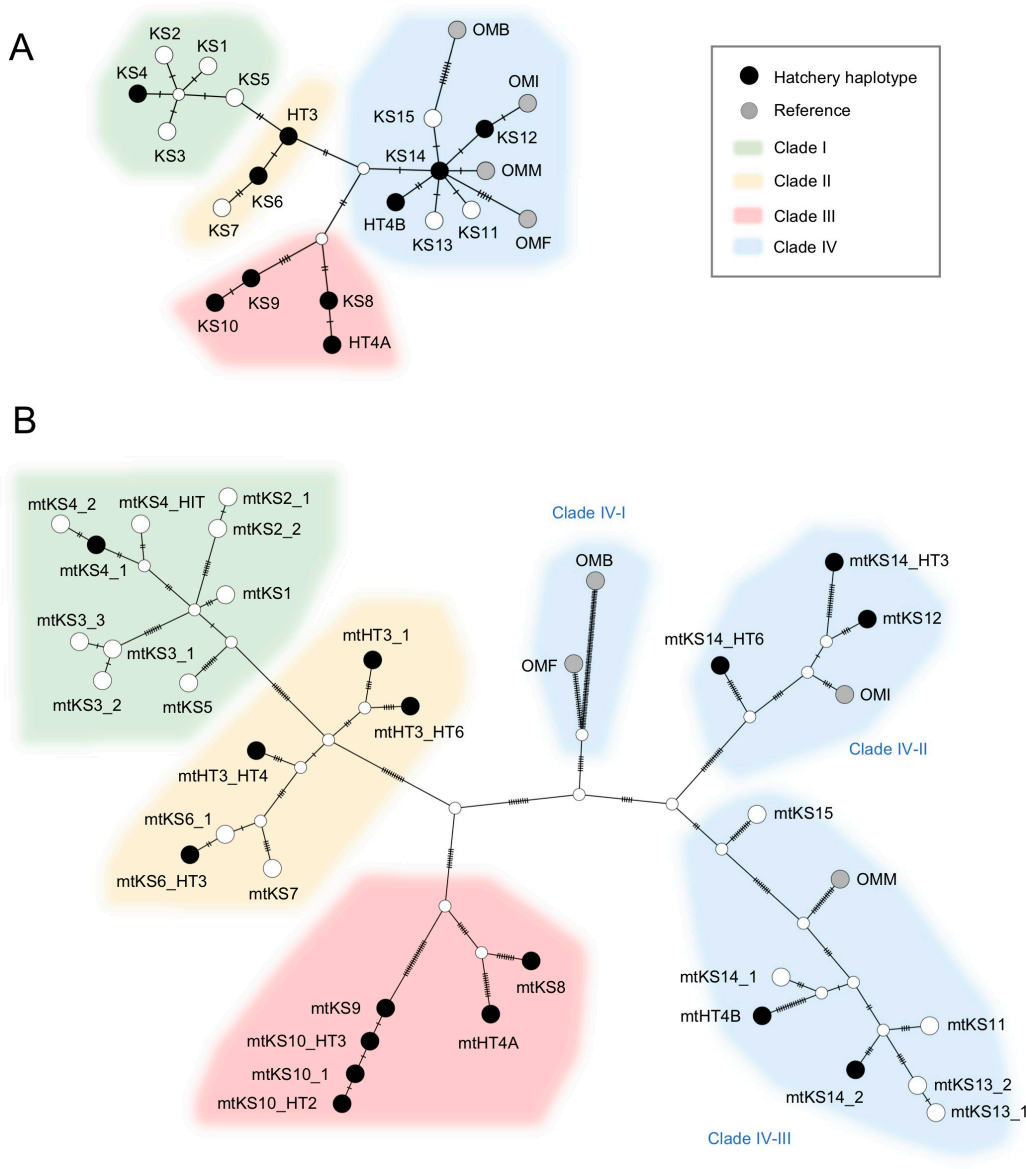
TCS network trees of *ND5* haplotypes and *MT* haplotypes obtained in this study. TCS network trees of (A) *ND5* haplotypes and (B) *MT* haplotypes are shown. Each dash represents one single nucleotide difference between two neighboring haplotypes. Reference information (abbreviations and sequence accession numbers) are as follows: OMM, *O*. *m*. *masou* (NC_008747); OMI, *O*. *m*. *ishikawae* (DQ_864464); OMF, *O*. *m*. *formosanus* (DQ_858456); OMB, *O*. *m*. Biwa subsp. (Biwa salmon) (EF_105342).

We analyzed the relationships among haplotypes by using the TCS program (see Materials and Methods). The results showed a network of *ND5* haplotypes (*ND5*-haplotype network) comprising four clades (Fig 2A). Analysis of *MT* haplotype relationships showed a detailed network (*MT*-haplotype network) (Fig 2B). Clades I–III in the *ND5*-haplotype network were similar to those in the *MT*-haplotype network. Clade IV of the *ND5*-haplotype network included other *O*. *masou* subspecies and formed a star-like topology radiating from haplotype KS14, whereas clade IV of the *MT*-haplotype network was divided into three sub-clades (Clade IV-I–IV-III), and the reference sequences of *O*. *m*. Biwa subsp. (Biwa salmon) (Accession No.: EF_105342) and *O*. *m*. *formosanus* (Accession No.: DQ_858456) belonged to clade IV-I. In both the *ND5-* and *MT*-haplotype networks, all haplotypes in clade III were hatchery haplotypes. Additionally, clade IV-II of the *MT*-haplotype network was also all hatchery haplotypes. In clade I of the *MT*-haplotype network, mtKS4_1, which was detected at sKFR (original river: Ishido tributary in the Hitotsuse River system), was in the same branch as mtKS4_HIT, which was detected at sSRF (original river: other tributary in the Hitotsuse River system).

We compared the haplotypes and cladal relationships obtained in this study with those obtained using sequencing data of a 561 bp region in *ND5* in the reports by Kitanishi et al. [9] and Yu et al. [13] (S3 Table and S1 Fig). KS1, KS2, and KS13, were new haplotypes in this region of *ND5* in the current study. Clades I, II, III, and IV in the current study appear to correspond to clades 1–3, 1–6, 2–3, and 2–1, reported by Yu et al. [13], respectively (S3 Table).

## Discussion

We obtained 32 *MT* haplotypes of *O*. *m*. *masou* from the Kase River system, hatcheries, and a control group by whole-mitogenome sequencing in this study. This analysis clearly identified hatchery haplotypes in this species in the Kase River system, like fingerprints. Sites contaminated with fish with hatchery *MT* haplotypes were clarified, suggesting that informal stocking was widely done across the study area. In the Kase River system, we obtained 21 *MT* haplotypes, six of which matched the haplotypes of the reference hatcheries (Fig 1 and Table 4). In pre-information, five sites had a stocking record (Table 1; bold letters, Fig 1), however, these hatchery haplotypes were detected in 13 of 17 sites in this river system (Fig 1). Hatchery haplotypes were also distributed upstream of waterfalls and artificial barriers that were built before stocking become popular in the Kase River system in the early 1970s (Fig 1) [30]. *Oncorhynchus masou masou* has a high homing tendency for the natal stream, and the dispersal of anadromous females and river-resident individuals is low [31–33]. Hence, we consider that hatchery haplotypes in each site were derived from artificially stocked fish rather than natural dispersal. AMOVA suggested that these stocked hatchery haplotypes reduce the genetic variation among populations in this river system (Table 5).

Hatchery haplotype mtKS4_1 in the Kase River system was inferred to be derived from sKFR and spread from the hatchery sHT1 on the Kase River system. Partial mitogenome analysis showed that the *ND5* haplotype KS4 was detected widely and frequently across the Kase River system (Table 3). However, with whole-mitogenome analysis, fish with this haplotype could be divided into three *MT* haplotypes, mtKS4_1, mtKS4_2, and mtKS4_HIT. In the Kase River system, whole-mitogenome sequence of analyzed samples with *ND5* haplotype KS4 displayed all the *MT* haplotype mtKS4_1, except a sample at St8 with *MT* haplotype mtKS4_2 (Table 4). Prior to this study, we had obtained the information that the fish of hatchery sHT1 originated from sKFR. The result of whole-mitogenome analysis was consistent with this information, in that whole-mitogenome sequencing of samples with the *ND5* haplotype KS4 from sHT1 and sKFR displayed the *MT* haplotype mtKS4_1 (Fig 1 and Table 1). These results allowed us to estimate of the origin of this haplotype. We considered that the *MT* haplotype mtKS9 of hatchery sHT1 may be a strain that was subsequently added to this hatchery. However, it is unknown whether the *MT* haplotype mtKS9 detected in the field is from sHT1, because fish with this haplotype are also used in hatcheries sHT3 and sHT5.

We predict that habitats without hatchery haplotypes occur in three sites (St1, St12, and St14). These areas might be candidates for conservation, because indigenous fish likely remain there. Although no hatchery haplotypes were detected in four sites (St1, St4, St12, and St14; Fig 1 and Table 4), we consider that the mtKS15 haplotype detected at St4 could be a stocked hatchery haplotype that could not be detected in this study, because (a) St4 has a stocking record (Table 1), and (b) mtKS15 was also detected at sites St13 and St16, which are geographically distant from St4, as observed with other hatchery *MT* haplotypes (Fig 1 and Table 4). Hybridization with invading hatchery male fish cannot be checked by mitogenome analysis alone. Therefore, whether hybridization with hatchery fish occurred at these locations will need to be confirmed in further studies by nuclear genome analysis, such as microsatellite analysis [19, 34]. Additionally, this suspicious haplotype might be resolved by accumulating whole-mitogenome sequence data of hatcheries and field samples from several other areas in Japan in future studies.

Indigenous haplotypes must be among the “other haplotypes”. In this study, the proportion of “other haplotypes” was highest in clade I (68.8%), possibly suggesting that clade I is the main population of indigenous *O*. *m*. *masou* in the Kase River system (Tables 3 and 4, and Fig 2). As shown in S1 Fig, we obtained three new haplotypes, KS1, KS2, and KS13, in the *ND5* 561 bp region in this study. KS1 and KS2 are not hatchery haplotypes and belong to clade I (Fig 2). The only haplotype corresponding to clade I in previous studies is H22 (S3 Table and S1 Fig) [13], however this haplotype was not detected in the north of Japan’s Hokkaido Island and was detected at a low rate in Honshu Island and in Korea [9, 13]. These results may suggest that the clade composition differs between the Kase River system on Kyushu Island and northern Japan. A recent study using a cytochrome-*b* gene (1141 bp region) in *O*. *masou* in the northwestern Pacific including southern Kyushu Island suggested the possible existence of a specific clade comprised of only *O*. *m*. *masou* in Kyushu Island (Group D in [15]). Further study might be necessary to assess whether our clade I haplotypes are related to Group D haplotypes in that report.

This study suggested that the whole-mitogenome analysis can detect a subtle divergence of the mitogenome in this species. As a notable example, the *ND5* haplotype detected at the control site, prohibited fishing area sSRF, was identical to that detected at hatcheries sHT1 and sKFR (KS4; Table 3); however, the *MT* haplotype detected at sSRF (i.e., mtKS4_HIT) and that detected at the above hatcheries (i.e., mtKS4_1) differed by four nucleotides (Table 4 and S2 Table), and they diverged from the same node in the *MT*-haplotype network (Fig 2B). Fish in the sHT1 hatchery are known to originate from sKFR, which in turn sourced fish from the Ishido tributary of the Hitotsuse River system. Site sSRF is in another tributary of the same Hitotsuse River system (Table 1). Therefore mtKS4_HIT might be an indigenous haplotype at sSRF, and it is possible that fish with these two haplotypes diverged from a common ancestor in the Hitotsuse River system and accumulated genetic mutations at each site due to their homing ability and/or being landlocked [33, 35]. In the Kase River system, such subtle gene divergence was primarily observed “other haplotypes” in clade I (the clade in which main population of indigenous are inferred to belong) (Table 4 and Fig 2B). These haplotypes might be important for this river system because they may reflect its history. Further investigation might be necessary in the future whether many of these *MT* haplotypes are unique to each tributary because whole-mitogenome analysis was performed on only a few samples in each site.

This study also clarified that some reared strains were shared by multiple hatcheries: e.g., hatchery *MT* haplotype mtKS8 was detected at sHT3, sHT4, and sHT6; hatchery *MT* haplotype mtKS9 was detected at sHT1, sHT3, and sHT5 (Fig 1 and Table 4). Hatcheries sHT2-HT6 are used by fisheries cooperatives, fishing clubs, and/or many individuals, and are likely used to stock various rivers (e.g., [16, 27]). It is of concern that stocking of multiple rivers could cause loss of genetic diversity in this species among rivers [2, 5, 36]; the results of our AMOVA of the Kase River system are consistent with this notion. The 16 hatchery *MT* haplotypes identified in this study might also contribute to resource management in other rivers because, as mentioned above, the several reared strains that shared multiple hatcheries were likely also used to stock other rivers.

In conclusion, whole-mitogenome analysis helped the genetic study of *O*. *m*. *masou*, and provided useful information that was not available from conventional partial analysis of the mitogenome. The findings of this study reveal the reality of fish stocking in the Kase River system and suggest a critical situation—loss of indigenous habitat and genetic variation diversity—caused by stocking in this river system. Further investigation of hybridization with invading hatchery fish would require nuclear genome analysis. In addition, it will be necessary to pay attention to genetic fluctuations so that the indigenous resources of this river system will not deteriorate further by additional stocking, increasing the frequency of hatchery haplotypes and/or the disappearance of “other haplotypes (especially clade I haplotypes that inferred to be main indigenous population in this river system)”. Whole-mitogenome analysis could not be performed on all samples in this study, so it may be necessary to obtain additional genetic information for some purposes; however, the obtained data will contribute as basic information on *O*. *m*. *masou*. For example, although mitogenome analysis cannot check for hybridization with hatchery fish, the SNP data obtained here for *O*. *m*. *masou* (S2 Table) could be utilized as basic information when designing a detection system for target haplotypes; it might be useful for early resource management of this river and other river systems, such as monitoring of informal stocking and stocked hatchery fishes and/or putative indigenous resources by eDNA analysis [20, 37].

## Supporting information

S1 Fig TCS network trees showing the relationship between data from past studies and the present study. Double circled haplotypes are new haplotypes obtained by this study. Colors show the clades corresponded to this study in Fig 2. For details showing the collation of haplotypes of past studies and this study see S3 Table.
(PDF)

S1 Table List of GenBank accession numbers deposited in this study. (PDF)

S2 Table List of *MT* haplotype SNP sequences for the reference sequence (*O*. *m*. *masou* [NC_008747]). Dots represent the same nucleotide as the reference sequence. “−” represents a gap site. Asterisks show a nucleotide differing only with the reference sequence. The gray column indicates the *ND5* sequenced region.
(XLSX)

S3 Table Collation of haplotypes determined in past studies and this study. Haplotype H1–H23 data are from Kitanishi et al. [9] and Yu et al. [13]. OMM:*O*. *m*. *masou* (NC_008747). OMI: *O*. *m*. *ishikawae* (DQ_864464). OMF: *O*. *m*. *formosanus* (DQ_858456). OMB: *O*. *m*. Biwa subsp. (Biwa salmon) (EF_105342).
(PDF)

## References

[1] Lever C. Naturalized Fishes of the World. London: Academic Press; 1996 Available from: https://books.google.co.jp/books?id=Q5QWAQAAIAAJ

[2] Hindar K, Ryman N, Utter F. Genetic Effects of Cultured Fish on Natural Fish Populations. Can J Fish Aquat Sci. 1991;48: 945–957.

[3] Rhymer JM, Simberloff D. Extinction by hybridization and introgression. Annu Rev Ecol Syst. 1996;27: 83–109. 10.1146/annurev.ecolsys.27.1.83

[4] Araki H, Berejikian BA, Ford MJ, Blouin MS. Fitness of hatchery-reared salmonids in the wild. Evol Appl. 2008;1: 342–355. 10.1111/j.1752-4571.2008.00026.x 25567636 PMC3352433

[5] Finnegan AK, Stevens JR. Assessing the long-term genetic impact of historical stocking events on contemporary populations of Atlantic salmon, *Salmo salar*. Fish Manag Ecol. 2008;15: 315–326. 10.1111/j.1365-2400.2008.00616.x

[6] Hansen MM, Fraser DJ, Meier K, Mensberg KLD. Sixty years of anthropogenic pressure: A spatio-temporal genetic analysis of brown trout populations subject to stocking and population declines. Mol Ecol. 2009;18: 2549–2562. 10.1111/j.1365-294X.2009.04198.x 19457206

[7] Kato F. Life histories of masu and amago salmon (*Oncorhynchus masou* and *Oncorhynchus rhodurus*) In: Groot C, Margolis L, editors. Pacific Salmon Life Histories. Vancouver: University of British Columbia Press; 1991 pp. 448–520. Available from: http://ci.nii.ac.jp/naid/10008268078/en/

[8] Ueda H. Sensory mechanisms of natal stream imprinting and homing in *Oncorhynchus* spp. J Fish Biol. 2019;95: 293–303. 10.1111/jfb.13775 30101534

[9] Kitanishi S, Edo K, Yamamoto T, Azuma N, Hasegawa O, Higashi S. Genetic structure of masu salmon (*Oncorhynchus masou*) populations in Hokkaido, northernmost Japan, inferred from mitochondrial DNA variation. J Fish Biol. 2007;71: 437–452. 10.1111/j.1095-8649.2007.01689.x

[10] Mayama H. *Oncorhynchus masou masou* ecological notes. Fish Egg. 1990;159: 7–21 (in Japanese). Available from: http://salmon.fra.affrc.go.jp/kankobutu/tech_repo/fe02/fishandegg159_p07-21.pdf

[11] Noguchi D, Taniguch N. Studies on the genetic diversity of wild populations by microsatellite of masu *Oncorhynchus* DNA markers. Aquac Sci. 2007;55: 521–527 (in Japanese with English summary). 10.10233/aquaculturesci1953.55.521

[12] Kitanishi S, Yamamoto T, Urabe H, Shimoda K. Hierarchical genetic structure of native masu salmon populations in Hokkaido, Japan. Environ Biol Fishes. 2018;101: 699–710. 10.1007/s10641-018-0730-6

[13] Yu J, Azuma N, Yoon M, Brykov V, Urawa S, Nagata M, et al Population genetic structure and phylogeography of masu salmon (*Oncorhynchus masou masou*) inferred from mitochondrial and microsatellite DNA analyses. Zoolog Sci. 2010;27: 375–385. 10.2108/zsj.27.375 20443684

[14] Yamamoto S, Morita K, Kikko T, Kawamura K, Sato S, Gwo J-C. Phylogeography of a salmonid fish, masu salmon *Oncorhynchus masou* subspecies-complex, with disjunct distributions across the temperate northern Pacific. Freshw Biol. 2019; 1–18. 10.1111/fwb.13460

[15] Iwatsuki Y, Ineno T, Tanaka F, Tanaka K. The southernmost population of *Onchorhynchus masou* masou from Kyushu Island, Japan and gross genetic structure of the *O. masou* complex from the northwestern Pacific. In: Gwo J-C, Shieh Y-T, Burridge CP, editors. Proceedings of International Symposium on the 100th Anniversary of the discovery of Formosa landlocked salmon Bull Natl Taiwan Museum. National Taiwan Museum; 2019. pp. 101–119.

[16] Ineno T, Taguchi T. A study on the genetic diversity of inland waters fish and shellfish. Bull MiyazakiPref Fish Exp Stn. 2007;1: 311–317 (in Japanese). Available from: http://www.mz-suishi.jp/cgi-bin/upload22/0232_Taro_0404.pdf

[17] Cann RL, Stoneking M, Wilson AC. Mitochondrial DNA and human evolution. Nature. 1987;325: 31–36. 10.1038/325031a0 3025745

[18] Forgacs D, Wallen RL, Dobson LK, Derr JN. Mitochondrial Genome Analysis Reveals Historical Lineages in Yellowstone Bison. PLoS One. 2016;11: e0166081 Available from: 10.1371/journal.pone.0166081 27880780 PMC5120810

[19] Fraser DJ, Bernatchez L. Adaptive evolutionary conservation: Towards a unified concept for defining conservation units. Mol Ecol. 2001;10: 2741–2752. 10.1046/j.1365-294X.2001.t01-1-01411.x 11903888

[20] Rees HC, Maddison BC, Middleditch DJ, Patmore JRM, Gough KC. REVIEW The detection of aquatic animal species using environmental DNA—a review of eDNA as a survey tool in ecology. J Appl Ecol. 2014;51: 1450–1459. 10.1111/1365-2664.12306

[21] Miya M, Sato Y, Fukunaga T, Sado T, Poulsen JY, Sato K, et al MiFish, a set of universal PCR primers for metabarcoding environmental DNA from fishes: Detection of more than 230 subtropical marine species. R Soc Open Sci. 2015;2 10.1098/rsos.150088 26587265 PMC4632578

[22] Bourret V, Albert V, April J, Côté G, Morissette O. Past, present and future contributions of evolutionary biology to wildlife forensics, management and conservation. Evol Appl. 2020; 1–15. 10.1111/eva.12977 32684967 PMC7359848

[23] Miya M, Nishida M. Use of mitogenomic information in teleostean molecular phylogenetics: a tree-based exploration under the maximum-parsimony optimality criterion. Mol Phylogenet Evol. 2000;17: 437–455. 10.1006/mpev.2000.0839 11133198

[24] Shamblin BM, Bjorndal KA, Bolten AB, Hillis-Starr ZM, Lundgren I, Naro-Maciel E, et al Mitogenomic sequences better resolve stock structure of southern Greater Caribbean green turtle rookeries. Mol Ecol. 2012;21: 2330–2340. 10.1111/j.1365-294X.2012.05530.x 22432442

[25] Feutry P, Kyne PM, Pillans RD, Chen X, Marthick JR, Morgan DL, et al Whole mitogenome sequencing refines population structure of the Critically Endangered sawfish *Pristis pristis*. Mar Ecol Prog Ser. 2015;533: 237–244. 10.3354/meps11354

[26] Bishop CR, Hughes JM, Schmidt DJ. Mitogenomic analysis of the Australian lungfish (*Neoceratodus forsteri*) reveals structuring of indigenous riverine populations and late Pleistocene movement between drainage basins. Conserv Genet. 2017;19: 587–597. 10.1007/s10592-017-1034-7

[27] Kimoto K, Mekata T, Takahashi H, Nagasawa K. Genetic structure of the amago and iwame forms of the red-spotted masu salmon *Oncorhynchus masou ishikawae* in the upper Ono River, northeastern Kyushu, southern Japan. Aquacult Sci. 2015;63: 299–309. 10.10233/aquaculturesci.63.299

[28] Excoffier L, E L Lischer H. ARLEQUIN suite ver 3.5: a new series of programs to perform population genetics analyses under Linux and Windows. Molecular ecology resources. 2010 10.1111/j.1755-0998.2010.02847.x 21565059

[29] Leigh JW, Bryant D. POPART: Full-feature software for haplotype network construction. Methods Ecol Evol. 2015;6: 1110–1116. 10.1111/2041-210X.12410

[30] Kimura S. *Oncorhynchus masou masou* in Kyushu Ialand, specially reference to its spawning behavior. Animals of Kyusyu and Okinawa, 2 Nishinippon Shinbunsha; 1976 pp. 36–63 (in Japanese).

[31] Miyakoshi Y, Takahashi M, Ohkuma K, Urabe H, Shimoda K, Kawamuka H. Homing of masu salmon in the tributaries of the Shiribetsu River evaluated by returns of marked fish. Sci Rep Hokkaido Fish Res Inst. 2012;81: 125–129 (in Japanese with English summary). Available from: https://www.hro.or.jp/list/fisheries/marine/att/81-miyakoshi1.pdf

[32] Ueda H. Physiological mechanisms of imprinting and homing migration in Pacific salmon *Oncorhynchus* spp. J Fish Biol. 2012;81: 543–558. 10.1111/j.1095-8649.2012.03354.x 22803723

[33] Kitanishi S, Yamamoto T, Koizumi I, Dunham JB, Higashi S. Fine scale relationships between sex, life history, and dispersal of masu salmon. Ecol Evol. 2012;2: 920–929. 10.1002/ece3.228 22837837 PMC3399158

[34] Toews DPL, Brelsford A. The biogeography of mitochondrial and nuclear discordance in animals. Mol Ecol. 2012;21: 3907–3930. 10.1111/j.1365-294X.2012.05664.x 22738314

[35] Kitanishi S, Yamamoto T, Higashi S. Microsatellite variation reveals fine-scale genetic structure of masu salmon, *Oncorhynchus masou*, within the Atsuta River. Ecol Freshw Fish. 2009;18: 65–71. 10.1111/j.1600-0633.2008.00325.x

[36] Lodge DM. Biological Invasions—lessons for ecology. Trends Ecol Evol. 1993;8: 133–137. 10.1016/0169-5347(93)90025-K 21236129

[37] Tsuji S, Maruyama A, Miya M, Ushio M, Sato H, Minamoto T, et al Environmental DNA analysis shows high potential as a tool for estimating intraspecific genetic diversity in a wild fish population. Mol Ecol Resour. 2020; 1–11. 10.1111/1755-0998.13125 32293104

