# Supplementary figures and images for: Fingerprinting of hatchery haplotypes and acquisition of genetic information by whole-mitogenome sequencing of masu salmon, Oncorhynchus masou masou, in the Kase River system, Japan
Source: PLoS One. 2020 Nov 4;15(11):e0240823. doi: 10.1371/journal.pone.0240823 (PMC7641346; doi:10.1371/journal.pone.0240823)

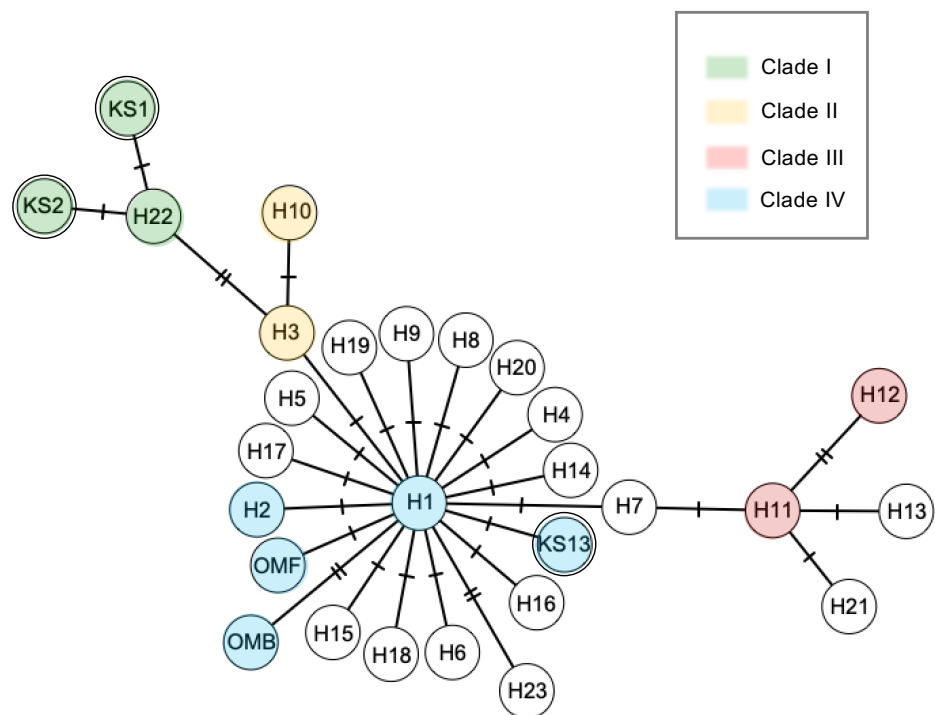

**S1 Fig.**

Supplement: S1 Fig — Double circled haplotypes are new haplotypes obtained by this study. Colors show the clades corresponded to this study in Fig 2. For details showing the collation of haplotypes of past studies and this study see S3 Table. (PDF) [file pone.0240823.s001.pdf]
