# Supplementary material for: Fingerprinting of hatchery haplotypes and acquisition of genetic information by whole-mitogenome sequencing of masu salmon, Oncorhynchus masou masou, in the Kase River system, Japan
Source: PLoS One. 2020 Nov 4;15(11):e0240823. doi: 10.1371/journal.pone.0240823 (PMC7641346; doi:10.1371/journal.pone.0240823)
Supplement: S1 Table — (PDF) [file pone.0240823.s002.pdf]

S1 Table. List of GenBank accession numbers deposited in this study

Haplotype name	DDBJ/EMBL/GenBank no.
ND5 haplotype	
KS1	LC361126
KS2	LC361127
KS3	LC361128
KS4	LC361129
KS5	LC361130
KS6	LC361131
KS7	LC361132
KS8	LC361133
KS9	LC361134
KS10	LC361135
KS11	LC361136
KS12	LC361137
KS13	LC361138
KS14	LC361139
KS15	LC361140
HT3	LC361144
HT4A	LC361145
HT4B	LC361146
MT haplotype	
mtKS1	LC381866
mtKS2_1	LC381867
mtKS2_2	LC381868
mtKS3_1	LC381869
mtKS3_2	LC381870
mtKS3_3	LC381871
mtKS4_1	LC381872
mtKS4_2	LC381873
mtKS4_HIT	LC381874
mtKS5	LC381876
mtKS6_1	LC381877
mtKS6_HT3	LC381878
mtKS7	LC381879
mtKS8	LC381880
mtKS9	LC381881
mtKS10_1	LC381884
mtKS10_HT2	LC381885
mtKS10_HT3	LC381886
mtKS11	LC381887
mtKS12	LC381888
mtKS13_1	LC381889
mtKS13_2	LC381890
mtKS14_1	LC381891
mtKS14_2	LC381892
mtKS14_HT3	LC381896
mtKS14_HT6	LC381897
mtKS15	LC381898
mtHT3_1	LC381903
mtHT3_HT4	LC381904
mtHT3_HT6	LC381905
mtHT4A	LC381908
mtHT4B	LC381909
