# Supplementary material for: Fingerprinting of hatchery haplotypes and acquisition of genetic information by whole-mitogenome sequencing of masu salmon, Oncorhynchus masou masou, in the Kase River system, Japan
Source: PLoS One. 2020 Nov 4;15(11):e0240823. doi: 10.1371/journal.pone.0240823 (PMC7641346; doi:10.1371/journal.pone.0240823)
Supplement: S3 Table — Haplotype H1–H23 data are from Kitanishi et al. [9] and Yu et al. [13]. OMM:O. m. masou (NC_008747). OMI: O. m. ishikawae (DQ_864464). OMF: O. m. formosanus (DQ_858456). OMB: O. m. Biwa subsp. (Biwa salmon) (EF_105342). (PDF) [file pone.0240823.s004.pdf]

S3 Table. Collation of haplotypes determined in past studies and this study

Past study			This study	
**Haplotype**	**GenBank Accession no.**	**Clade (1-step, 2-step)**	**Corresponding haplotypes**	**Clade**
H1	ab252719	1-1, 2-1	KS11, KS12, KS14, KS15, HT4B, (OMM, OMI)	IV
H2	ab252720	1-1, 2-1		
H3	ab252721	1-6, 2-2	HT3	II
H4	ab252722	1-1, 2-1		
H5	ab252723			
H6	ab252724			
H7	ab252725	1-4, 2-3		
H8	ab252726	1-1, 2-1		
H9	ab252727			
H10	ab252728	1-6, 2-2	KS6, KS7	II
H11	ab252729	1-4, 2-3	KS8, HT4A	III
H12	ab252730	1-5, 2-3	KS9, KS10	III
H13	ab252731	1-4, 2-3		
H14	ab469790	1-1, 2-1		
H15	ab469791	1-1, 2-1		
H16	ab469792	1-1, 2-1		
H17	ab469793	1-1, 2-1		
H18	ab469794	1-1, 2-1		
H19	ab469795	1-1, 2-1		
H20	ab469796	1-1, 2-1		
H21	ab469797	1-4, 2-3		
H22	ab469798	1-3, 2-2	KS3, KS4, KS5	I
H23	ab469799	1-2, 2-1		
-	-		KS1	I
-	-		KS2	I
-	-		KS13	IV
-	-		(OMF)	IV
-	-		(OMB)	IV

Haplotype H1–H23 data are from Kitanishi et al. [9] and Yu et al. [13]. OMM: *O. m. masou* (NC_008747). OMI: *O. m. ishikawae* (DQ_864464). OMF: *O. m. formosanus* (DQ_858456). OMB: *O. m* . Biwa subsp. (Biwa salmon) (EF_105342).
